# Maternal Dietary Patterns and Gestational Diabetes Risk: A Case-Control Study

**DOI:** 10.1155/2017/5173926

**Published:** 2017-12-06

**Authors:** Fatemeh Sedaghat, Mahdieh Akhoondan, Mehdi Ehteshami, Vahideh Aghamohammadi, Nila Ghanei, Parvin Mirmiran, Bahram Rashidkhani

**Affiliations:** ^1^Department of Basic Medical Sciences, Faculty of Nutrition Sciences and Food Technology, National Nutrition and Food Technology Research Institute, Shahid Beheshti University of Medical Sciences, Tehran, Iran; ^2^Department of Community Nutrition, Faculty of Nutrition Sciences and Food Technology, National Nutrition and Food Technology Research Institute, Shahid Beheshti University of Medical Sciences, Tehran, Iran; ^3^Department of Paramedical Sciences, Ahvaz Jundishapur University of Medical Sciences, Ahvaz, Iran; ^4^Nutrition and Endocrine Research Center, Research Institute for Endocrine Sciences, Shahid Beheshti University of Medical Sciences, Tehran, Iran

## Abstract

**Background:**

Maternal dietary patterns play an important role in the progress of gestational diabetes mellitus (GDM). The aim of the present study was to explore this association.

**Method:**

A total of 388 pregnant women (122 case and 266 control) were included. Dietary intake were collected using a food frequency questionnaire (FFQ). GDM was diagnosed using a 100-gram, 3-hour oral glucose tolerance test. Dietary pattern was identified by factor analysis. To investigate the relation between each of the independent variables with gestational diabetes, the odds ratio (OR) was calculated.

**Results:**

Western dietary pattern was high in sweets, jams, mayonnaise, soft drinks, salty snacks, solid fat, high-fat dairy products, potatoes, organ meat, eggs, red meat, processed foods, tea, and coffee. The prudent dietary pattern was characterized by higher intake of liquid oils, legumes, nuts and seeds, fruits and dried fruits, fish and poultry whole, and refined grains. Western dietary pattern was associated with increased risk of gestational diabetes mellitus before and after adjustment for confounders (OR = 1.97, 95% CI: 1.27–3.04, OR = 1.68, 95% CI: 1.04–2.27). However, no significant association was found for a prudent pattern.

**Conclusion:**

These findings suggest that the Western dietary pattern was associated with an increased risk of GDM.

## 1. Introduction

Gestational diabetes mellitus (GDM), defined as any degree of glucose intolerance with onset during pregnancy, is a common complication, and its prevalence ranges between 7 and 14% worldwide [1].

Incidence of GDM among Iranian women living in urban areas has been reported similar to developing countries [2–5]. However, with the prevalence of type II diabetes increasing across the world and considering that the prevalence of GDM is thought to shadow that of type II diabetes, we expect to see a rise in GDM incidence during the coming years [3].

Thus, it is crucial to detect modifiable risk factors that may contribute to the prevention of GDM. Food and dietary factors have been reported to affect glucose homeostasis, and diet may be associated with GDM risk factors [6, 7]. Several studies found a positive association between GDM risk and intake of total fat [8, 9], saturated fat [10, 11], and an inverse relation between the risk of GDM and polyunsaturated fat [10, 12]. In contrast, no significant association was found between total dietary, saturated, and polyunsaturated fat intake and the risk of GDM in another study [13].

However, most previous studies have traditionally focused on the role of the single food items [14], nutrients [15, 16], or food groups [17, 18]. Considering the limitations of these kinds of studies, such as neglecting the interactions and synergistic effects of nutrients [19], detecting dietary patterns offers a comprehensive and complimentary method in investigating the relationship between diet and disease risk [20]. Dietary patterns are population specific and are influenced by sociocultural factors and food availability [20]. The results of recent systematic reviews showed that adherence to a healthier dietary pattern, like Mediterranean dietary pattern, and reducing the intake of sugar sweetened cola, potatoes, fatty foods, sweets, and food with high heme iron content can decrease the incidence of GDM, especially in women at higher risk and before getting pregnant [7, 21]. However, the majority of the studies to date have been conducted in western populations [17, 22, 23]. Conversely, studies investigating dietary patterns in relation to GDM in Asian women, who are at a much greater risk of developing GDM compared to others ethnics, are sparse [24, 25]. Due to the growing trend of gestational diabetes and a shortage of research on the effect of dietary patterns on the prevention of GDM in Asian women, this study aimed to investigate the association between prepregnancy dietary patterns and risk of GDM in Iranian women.

## 2. Materials and Methods

### 2.1. Participants

This hospital-based case-control study was conducted in Tehran, a province of Iran at high-risk of diabetes. Cases (*n* = 123) with pregnant women aged 18–40 years who visited major general hospitals in different regions of Tehran (11 million inhabitants) were included. Pregnant women were screened for gestational diabetes between the 24th and 28th weeks of gestation with a 50 g, 1 hr glucose challenge test (GCT). If the screening test was positive (blood glucose greater than 130 mg per ml), diagnostic testing was performed using a 100 g, 3-hour oral glucose tolerance test (OGTT). Women meeting the Carpenter and Coustan criteria [26], fasting 5.3 mmol/l, 1 h 10.0 mmol/l, 2 h 8.6 mmol/l, and 3 h, 7.8 mmol/l, were diagnosed with GDM (any two values at or above established thresholds).

Controls were pregnant women (*n* = 268) whose GCT tests at 24–28 weeks of pregnancy were in the normal range. The exclusion criteria were multiple pregnancies, history of gestational diabetes or diabetes (prepregnancy), and undergoing a weight-reduction diet one year before pregnancy. Furthermore, controls were followed up until the end of pregnancy. If they developed GDM, they were excluded from the study. In this study, two controls were recruited within the same medical center for each case. Controls were matched to cases on age (within 5 years). During analysis, three patients (1 case and 2 controls) with extreme energy intakes that probably reflected careless completion of the dietary questionnaire (below or above the mean ± 3 SD for log-transformed calories; cutpoints: 244 kcal and 5284 kcal) were excluded. The final sample for statistical analysis was 122 cases and 266 controls.

Informed consent was obtained from each participant prior to enrollment. The study protocol was approved by the ethics committee at the National Nutrition and Food Technology Research Institute of Shahid Beheshti University of Medical Science.

### 2.2. Dietary Intake

One year before pregnancy, dietary intake of all participants were collected through validated semiquantitative food frequency questionnaires (FFQs) by trained interviewers. The FFQ consisted of 147 food items, including some of the most common Iranian meal recipes, and has been previously shown to be valid and reproducible for use in Iranian adults [27]. Subjects were asked to mention their consumption frequency of a given serving of each food item during the past year, on a daily, weekly, monthly, or yearly basis. Common household measures (measuring cups, spoons, and palm of hand) were used for better estimation of the real portion consumed by the subjects [28]. Portion sizes consumed from each food item were then converted to daily gram intake using the household scales [28]. In addition to the daily energy, macronutrient and micronutrient consumption for participants was calculated using the United States Department of Agriculture Food Composition Databases (USDA FCT). However, for some dairy products such as Kashk, wild plum, mint, sweet canned cherry, and sour cherry that are not listed in the USDA FCT, Iranian FCT was used alternatively [28].

### 2.3. Assessment of Nondietary Exposures

Information on age, prepregnancy weight, education level, socioeconomic status, cigarette smoking, family history of diabetes, and taking supplements were obtained from all cases and controls by trained professional interviewers using a questionnaire. The weight of each subject was measured with minimum clothing, and 100 g sensitivity and height was measured with using a nonstretch tape-meter fixed to a wall and was recorded to the nearest 0.5 cm. Body mass index (BMI) was computed subsequently by the formula (weight in kg)/(height in meter)^2^.

Physical activity level before pregnancy and average time per day spent on different intensity activities was evaluated using a validated self report-based questionnaire [29] and was expressed as metabolic equivalents hours/day (METs-h/d) in which nine different MET levels were ranged on a scale from sleep/rest (0.9 METs) to high-intensity physical activities (>6 METs). By multiplying the time spent on each activity level by the MET value of each activity, the MET-time for an activity was computed. Based on questionnaire data, we estimated a total activity score by adding the specific activities together.

### 2.4. Statistical Analysis

All analyses were performed using the Statistical Package for Social Sciences software version 16 (SPSS Inc., Chicago, IL, USA), and a two-sided *p* value < 0.05 was considered significant. The Kaiser–Meyer–Olkin test (KMO = 0.7) indicated adequacy of sampling. Bartlett's test of sphericity was significant (*p* < 0.001) indicating that factor analysis was suitable for the data.

For identifying the dietary patterns, food items obtained from the FFQ were categorized into 22 groups based on the similarity of nutrients (Table 1). Principal component analysis was used to identify major dietary patterns based on the 22 food groups. Two interpretable factors were retained based on the Scree test [19]; an orthogonal rotation procedure, the Varimax rotation, was then applied to simplify the factor structure facilitating interpretation. The labeling of derived factors was done on the basis of interpretation of the data and of the earlier literature. The factor score for each pattern was calculated by summing intakes of food groups weighted by factor loading, and individuals were assigned a factor score for each dietary pattern [19].

Two dietary pattern scores were divided into two categories based on the medians. To evaluate the differences in distribution of categorical variables, chi-square test was applied. To assess the differences in distribution of continuous variables across the dietary pattern score categories, independent sample *t*-test was used in case of normality, and Mann–Whitney test was used where the distribution of variables were not normal. Unconditional logistic regression was used in estimating odds ratio (OR) with 95% confidence interval (CI) after controlling for confounding variables (prepregnancy weight, gestational age, physical activity, family history of diabetes, housing ownership, and building area).

## 3. Results

Table 2 compares the characteristics of 122 cases and 266 controls. By design, age was similar in both groups (29.7 years versus 29.6 years in controls and cases, resp.). Cases had higher prepregnancy BMI and family history of diabetes compared to controls, while gestational age and physical activity were higher significantly in the control group (*p* < 0.001). Controls reported higher building area and housing ownership. No significant differences were observed for maternal age, energy intake, smoking status, and education status.

Factor-loading matrix for the 2 retained factors is shown in Table 3. Two dietary patterns were derived with eigenvalues above 2 from the scree plot, as well as factor loadings using factor analysis; these two patterns accounted for 19.4% of the total variation in food intakes. Dietary patterns were denoted “Western” and “prudent” according to previous studies. The “Western” dietary pattern had high positive factor loadings for sweet snacks, jam and tinned fruits, mayonnaise, sugar-sweetened beverages, salty snacks, solid fats, high-fat dairy, potatoes, organ meats, eggs, red and processed meat, and tea and coffee, as well as negative factor loading for low fat dairy, legumes, and whole grains. The “prudent” dietary pattern had high positive factor loadings for liquid oils, legumes, nuts and seeds, fruits and dried fruits, fish and poultry whole, and refined grains.

Characteristics of the study participants across median categories of the dietary pattern scores are shown in Table 4. BMI, family history of diabetes, parity, and energy intake was higher in pregnant women with higher scores of “Western” dietary pattern compared to those with lower scores (*p* < 0.05). Women with higher scores of the “prudent” dietary pattern had higher energy intake and education level compared to those with lower scores (*p* < 0.05).

The ORs and their 95% CI for GDM by the median of dietary pattern scores are displayed in Table 5. Risk of developing GDM among women in the second median of “Western” dietary pattern scores was higher compared to the first median (OR = 1.97, 95% CI: 1.27–3.04). After adjusting for prepregnancy weight, gestational age, physical activity, family history of diabetes, housing ownership, and building area, the association was still significant (OR = 1.68, 95% CI: 1.04–2.27). However, no significant association was found between “prudent” dietary pattern scores and risk of GDM.

## 4. Discussion

In the present study, we identified two distinct dietary patterns among participants: Western dietary pattern (high in sweets, jams, mayonnaise, soft drinks, salty snacks, solid fat, high-fat dairy products, potatoes, organ meat, eggs, red meat, processed foods, tea and coffee and low-fat dairy product, and whole grains) and prudent dietary pattern (which includes high intake of liquid oils, legumes, nuts and seeds, fruits and dried fruits, fish and poultry whole, and refined grains). Western dietary pattern was positively associated with an increased risk of gestational diabetes, after adjustment for potential confounders. However, there was no significant association between healthy dietary pattern and risk of GDM.

Healthy dietary patterns have consistently been associated with a reduced risk of type 2 diabetes mellitus (T2DM); however, few studies have investigated the association between prepregnancy dietary patterns and risk of GDM [17, 22, 23], and to our knowledge, only few has been conducted in Asian females [24, 25].

In a large prospective cohort study in 3063 pregnant Chinese women, no significant association was found between prudent pattern and GDM risk, while the sweets and seafood pattern was associated with an increased risk of GDM [25].

Our findings are further supported by the findings from the Nurses' Health Study II [17]. Women in the highest quintile of Western dietary pattern were associated with increased likelihood of GDM compared to those women in the lowest quintile [17].

Pregnancy is characterized by progressive hyperlipidemia, insulin resistance, and a deterioration of glucose tolerance as the pregnancy advances to the third trimester [30]. Prior evidence suggests that women who develop GDM have altered functions of *β*-cells and insulin resistance, compromising their capacity to deal with the metabolic challenges of pregnancy [17, 22, 30]. The Western dietary pattern and a dietary pattern similar to that were associated with a significant increase in fasting insulin and C-peptide levels and higher plasma glucose concentrations in healthy individuals [31].

Red and processed meats as one of the main components of the Western dietary pattern are sources of saturated fat, heme iron, nitrosamines, and other constituents which have been associated with beta cell damage, oxidative stress, and insulin resistance as well as incident GDM [17, 22]. Toxicity effects of nitrosamines which are produced by the reaction of nitrite (commonly used as a preservative in processed meats) with amine compounds has been reported in deterioration of beta cell function, increased lipid peroxidation and proinflammatory cytokine activation [32]. In addition, early limited N-nitrosodiethylamine (NDEA) exposures play critical roles in the pathogenesis of major insulin resistance diseases including T2DM in animal models [33]. Furthermore, advanced glycation end products (AGEs) produced during the heating process in red meat and high-fat products are suggested as another possible mediator of association between red and processed meat and GDM [34]. Moreover, it has been observed that high intakes of saturated and trans fatty acids by reducing insulin binding ability to its receptors and impairing glucose transport could be one of the important risk factors triggering GDM [21, 35].

Another potential explanation is related to a high consumption of sugar-sweetened beverages contributing to a high glycemic load (GL) diet leading to inflammation, insulin resistance, and impaired *β*-cell function [14, 30]. In addition, soft drinks contain caramel colouring which are rich in AGEs promoting inflammatory mediators that might be important in the development of diabetes, such as C-reactive protein and TNF-*α* [34]. It is also plausible that low intakes of whole grains in Western dietary pattern can contribute to GDM risk. Whole grains are high in insoluble fiber which delay gastric emptying and slow absorption of glucose, resulting in a small increment in insulin levels [6, 30].

On the other hand, some studies have shown an inverse relationship between a healthy dietary pattern and GDM [17]. The effects of a healthy dietary pattern on reducing risk of GDM may be due to a lower dietary energy density and glycemic load and higher amount of fruits and vegetables rich in antioxidants and phytochemicals, dietary fiber, and micronutrients such as magnesium and vitamin C [22, 30]. In our study, in line with some other studies [25], no significant association was found between healthy dietary pattern and gestational diabetes. This probably could be due to the presence of rice and refined grains in our healthy dietary pattern since rice and refined grains are Iranian staple foods [36]. It seems that the adverse effects of refined grains (high glycemic index and low fiber content) on glucose metabolism outweigh other benefits of healthy dietary pattern identified in our study.

Discrepancies between results of studies could be referring to differences in study design, sample size, food questionnaire, definition, and number of the food groups. Since dietary patterns reflect the culture, food preferences, and environmental factors (such as food availability), it can be expected that different dietary patterns are identified in different populations and time periods [19]. Dietary patterns derived from factor analysis capture the effect of the combination of many interacting and synergic foods facilitating the application of nutritional findings for public health recommendations and providing dietary guidelines which might be useful in the prevention of diseases. To our knowledge, the present study is the first one in a Middle-Eastern country to report the association between major dietary patterns and GDM. Studies in developing countries can provide unique opportunities to assess the relation between dietary patterns and GDM risk. Generally, where economic resources are severely restricted, food intake is strongly linked to income, so that even small economic differences are directly reflected in dietary intakes [37]. Other strengths of this analysis are that we were able to control for several potential confounders such as physical activity, prepregnancy BMI, and gestational age. In addition, high participation rate in this study, as 100% of cases and 97% of controls who were initially invited to participate in the research were retained in the final analyses, decreased the risk of selection bias. Since cases were more likely to have changed their diets due to the disease symptoms, the incident cases of GDM were registered in the present study to reduce the possibility of recall bias. One other strength is that the study was conducted in a province with a very high point prevalence of GDM [4].

The study has several limitations. First, because of the observational nature of the current study, we cannot rule out the possibility of residual confounding by unmeasured factors such as abnormal metabolic factors. However, significant associations remained after carefully controlling for major well-documented risk factors for GDM. Second, the results of factor analysis approach might be affected by several arbitrary but important decisions including the number of factors to extract, the components labelling, the method of rotation, and even their interpretation [38]. Third, since dietary intake was assessed using FFQ, measurement errors were unavoidable which can cause underestimation of associations. However, the FFQ applied in this study has relative good reproducibility and validity among the Iranian population [27], and we also excluded subjects overreporting their energy intakes. Fourth, due to the case-control study design, we cannot provide evidence of a causal relationship between prepregnancy dietary patterns and the risk for GDM.

Finally, the sample size of this study was small and the study was only conducted on women living in Tehran city. These could limit the generalization of study results to the entire Iranian population.

## 5. Conclusion

Overall, we found strong associations between Western dietary pattern and higher GDM risk, while no association was found between prudent pattern and GDM. However, case-control studies may prove an association but these do not demonstrate causation. As a result, these findings need to be confirmed in future prospective studies for etiological purposes to identify whether improving maternal's dietary pattern adherence before pregnancy is associated with a decreased risk of GDM.

## Figures and Tables

**Table 1:** Food groupings used in the dietary pattern analysis.

Food groups	Food items
Refined grains	White breads (lavash, baguettes), rice, pasta, noodles, biscuits
Fast foods	Sausages, bologna (beef), pizza
Potatoes	Potatoes (cooked and fried potatoes, French fries)
Salty snacks	Crackers, potato chips, corn puffs, pickled vegetables
Mayonnaise	Mayonnaise
Sugar sweetened beverages	Soft drinks, synthetic fruit juices
Eggs	Eggs
Vegetables	Lettuce, spinach, green leafy vegetables, onions, cucumber, turnip, cabbage, cauliflower, kale, eggplant, squash, celery, green pepper, garlic, mushrooms, green peas, green beans, broad beans, carrots, pumpkin, tomatoes, tomato sauce
Whole grains	Iranian dark breads, barley, corn, bulgur
Fruits and dried fruits	Apple, orange, tangerine, date, melon, watermelon and Persian melon, cantaloupe, banana, lemon, lime, apricots, grapes, cherries, strawberries, pomegranates, kiwi, grapefruit, persimmons, pear, peach, plums, nectarine, mulberry, fig, dried fruits, and natural fruit juices
Poultry and fish	Chicken, canned tuna fish, and every kind of fish
High fat dairy	Whole fat milk, cocoa milk, whole fat yoghurt, concentrated and creamy yoghurt, cream cheese, kashk, ice cream
Low-fat dairy	Low-fat milk, plain yoghurt, cheese, and yoghurt drink
Jam and tinned fruits	Jam, honey, and tinned fruits
Liquid oils	Vegetable oils, olives, and olive oil
Solid fats	Butter, margarine, cream, hydrogenated vegetable oils, and animal fats
Sweet snacks	Cakes, cookies, Iranian confectioneries (gaz, sohan, noghl), confections, sugars, chocolates, candies
Red meats	Beef, lamb, hamburger, ground meat
Organ meats	Liver, heart, kidney, tongue, feet, head, tripe, and brain
Tea and coffee	Tea, coffee
Nuts and seeds	Walnuts, almonds, hazelnuts, pistachios, peanuts, and roasted seeds
Legumes	Lentils, beans, chickpea, split peas, and soya beans

**Table 2:** Characteristics of patients in an Iranian GDM case-control study between 2009 and 2010, Tehran, Iran.

Characteristics	Controls	Cases	*p* value^a,b^
	*N* = 266	*N* = 122	
Age (years), mean ± SD	29.76 ± 4.26	29.64 ± 4.52	0.81
Gestational age (week), mean ± SD	31.19 ± 3.53	29.39 ± 4.74	<0.0001
Prepregnancy BMI (kg/m^2^), mean ± SD	24.64 ± 3.32	27.25 ± 3.82	<0.0001
Energy intake (kcal), mean ± SD	2672 ± 706	2818 ± 755	0.06
Physical activity (METs-h/d), mean ± SD	21.75 ± 26.37	12.92 ± 16.43	0.001
Family history of diabetes, *n* (%)	89 (33.46)	66 (54.55)	<0.0001
Supplement use a year before pregnancy, *n* (%)	84 (31.58)	45 (37.19)	0.28
Smoking exposure, *n* (%)	24 (9.02)	8 (6.61)	0.42
Education (%)			0.35
Illiterate and primary	29.7	24.4	
High school	41.3	48.8	
Diploma and over	29.0	26.8	
Housing ownership (yes), (%) History of abortion, *n* (%)	79.2	20.8	0.002
Building area (m^2^)	69	75	0.03

^a^Values are presented either as mean ± SD or *n* (%) for quantitative and qualitative variables, respectively; ^b^chi-square test and independent sample *t*-test were applied for categorical variables and continuous variables, respectively; *n* = 388.

**Table 3:** Factor loadings matrix for the major dietary patterns identified by factor analysis in an Iranian GDM case-control study between 2009 and 2010, Tehran, Iran (*n* = 388).^a^

Food groups	Western dietary pattern	Prudent dietary pattern
Refined grains	—	0.24
Fast foods	0.22	—
Potatoes	0.32	—
Salty snacks	0.44	—
Mayonnaise	0.43	0.27
Sugar sweetened beverages	0.40	—
Eggs	0.29	
Vegetables	—	—
Whole grains	−0.24	0.35
Fruits and dried fruits	—	0.51
Poultry and fish	—	0.43
High-fat dairy	0.34	—
Low-fat dairy	−0.21	—
Jam and tinned fruits	0.50	—
Liquid oils	—	0.69
Solid fats	0.38	—
Sweet snacks	0.48	—
Red and processed meats	0.23	0.21
Organ meats	0.31	—
Tea and coffee	0.25	—
Nuts and seeds	0.28	0.58
Legumes	−0.38	0.59
Explained variance (%)	10.3	8.9

^a^Values < 0.20 were excluded for simplicity.

**Table 4:** Participants' characteristics according to dietary pattern scores in an Iranian GDM case-control study between 2009 and 2010, Tehran, Iran.

Characteristics	Western dietary pattern^a^		Prudent dietary pattern^a^	
	Low	High	Low	High
Age (years)	29.6 ± 4.3	29.8 ± 4.4	29.6 ± 4.3	29.8 ± 4.4
*p* value^b^	0.5		0.8	
Prepregnancy BMI (kg/m^2^)	25.1 ± 3.5	25.9 ± 3.8	25.6 ± 3.9	25.4 ± 3.5
*p* value	0.02		0.54	
Gestational age (week)	30.5 ± 3.9	30.8 ± 4.1	30.5 ± 3.9	30.8 ± 4.1
*p* value	0.56		0.50	
Energy intake (kcal)	2489 ± 673	2984 ± 787	2275 ± 549	3194 ± 684
*p* value	<0.001		<0.001	
Family history of diabetes (%)	34.4	46.6	40.3	40.5
*p* value	0.02		0.85	
Smokers (%)	1.5	0	0.5	1.1
*p* value	0.24		0.62	
Physical activity (%)				
Low	49.0	51.0	54.6	45.4
High	51.0	49.0	45.4	54.6
*p* value	0.65		0.56	
Education (%)				
Illiterate and primary	5.5	8.3	9.7	4.1
High school	18.7	23.7	26.5	15.8
Diploma and over	75.8	68.0	63.8	80.1
*p* value	0.49		0.005	

^a^Low category comprises below median values, and high category corresponds to above median values; ^b^values are presented either as mean ± SD or (%) for quantitative and qualitative variables, respectively; *n* = 388.

**Table 5:** Unadjusted and adjusted odds ratios (OR) and 95% confidence intervals (CI) for gestational diabetes mellitus by median categories of dietary patterns in an Iranian GDM case-control study between 2009 and 2010, Tehran, Iran.

	Cases, *n* (%)	Controls, *n* (%)	Unadjusted OR (95% CI)	Adjusted OR^b^ (95% CI)
Western dietary pattern^a^				
Low	48 (39.0)	150 (55.8)	1.00 (reference)	1.00 (reference)
High	75 (61.0)	119 (44.2)	1.97 (1.27–3.04)	1.68 (1.04–2.72)
Prudent dietary pattern^a^				
Low	63 (51.2)	133 (49.4)	1.00 (reference)	1.00 (reference)
High	60 (48.8)	136 (50.6)	0.93 (0.60–1.42)	0.97 (0.61–1.56)

^a^Low category comprises below median values, and high category corresponds to above median values; ^b^adjusted for confounding variables (prepregnancy BMI, gestational age, physical activity, family history of diabetes, housing ownership, and building area); *n* = 388.
